# Risk algorithms that include pathology adjustment for HER2 amplification need to make further downward adjustments in likelihood scores

**DOI:** 10.1007/s10689-016-9942-0

**Published:** 2016-10-31

**Authors:** D. G. Evans, E. R. Woodward, S. J. Howell, S. Verhoef, A. Howell, F. Lalloo

**Affiliations:** 1 Manchester Centre for Genomic Medicine, St Mary’s Hospital, Manchester Academic Health Sciences Centre (MAHSC), Institute of Human Development, University of Manchester, Manchester, M13 9WL UK; 2 Genesis Breast Cancer Prevention Centre, University Hospital of South Manchester NHS Trust, Wythenshawe, Manchester, M23 9LT UK; 3 Manchester Centre for Genomic Medicine, St Mary’s Hospital, Manchester Academic Health Sciences Centre (MAHSC), Central Manchester University Hospitals NHS Foundation Trust, Manchester, M13 9WL UK; 4 Manchester Breast Centre, School of Molecular and Clinical Cancer Sciences, The University of Manchester, Manchester, M20 4BX UK; 5 Department of Medical Oncology, The Christie, Manchester, M20 4BX UK

**Keywords:** BRCA1, BRCA2, Breast cancer, HER2, Oestrogen, Manchester score

## Abstract

To assess the need for adjustment in the likelihood of germline *BRCA1/2* mutations in women with HER2+ breast cancers. We analysed primary mutation screens on women with breast cancer with unequivocal HER2 overexpression and assessed the likelihood of *BRCA1*/*BRCA2* mutations by age, oestrogen receptor status and Manchester score. Of 1111 primary BRCA screens with confirmed HER2 status only 4/161 (2.5%) of women with HER2 amplification had a *BRCA1* mutation identified and 5/161 (3.1%) a *BRCA2* mutation. The pathology adjusted Manchester score between 10 and 19% and 20%+ thresholds resulted in a detection rate of only 6.5 and 15% respectively. BOADICEA examples appeared to make even less downward adjustment. There is a very low detection rate of *BRCA1* and *BRCA2* mutations in women with HER2 amplified breast cancers. The Manchester score and BOADICEA do not make sufficient downward adjustment for HER2 amplification. For unaffected women, assessment of breast cancer risk and *BRCA1/2* probability should take into account the pathology of the most relevant close relative. Unaffected women undergoing mutation testing for *BRCA1/2* should be advised that there is limited reassurance from a negative test result if their close relative had a HER2+ breast cancer.

## Introduction

Although the pathology of breast cancers associated with *BRCA1* and to a lesser extent *BRCA2* is well documented [1–3], the likelihood of identifying a *BRCA1* or *BRCA2* mutation in HER2 amplified breast cancer is not well described. Most risk algorithms for assessing the likelihood of a *BRCA1/2* mutation were developed before the ability to adjust for pathology, particularly relevant for *BRCA1* where the majority of breast cancers are high grade and triple negative (estrogen receptor (ER), progesterone receptor (PR) and HER2 negative) [4–8]. The speed and reduced expense of modern *BRCA* mutation screening may lead to a perceived reduction in the importance of mutation likelihood assessment. However, difficulties in interpretation of mutation testing arise when a variant of uncertain significance (VUS) is found. Furthermore many countries still utilise likelihood thresholds for a sample to be tested, which in the UK remains at 10% [9]. Until there are licensed medications, approved by healthcare systems, for precision medicine approaches for breast cancer related to *BRCA1/2* such as PARPi, these thresholds are likely to remain. Knowing the a priori likelihood of an individual having a pathogenic mutation is helpful in interpreting VUS reports [3] that affect around 5% of tested individuals. More importantly, for those individuals testing negative for a *BRCA1/2* mutation screen who do not have cancer, and are from a family without testing of an affected member, it is not possible to assess the degree of reassurance of a negative test without knowledge of the likelihood that their affected relative was a *BRCA1* or *BRCA2* mutation carrier. Attempts have been made to incorporate breast pathology into risk algorithms such as the Manchester scoring system [10] and BOADICEA [11, 12] and these improve the accuracy of likelihood estimates and risk thresholds [10, 13]. Periodic revisions of these scoring systems are useful, as most ‘classical’ high penetrance BRCA mutation carrier families will have been identified, leaving less classical phenotypes to be uncovered. Therefore incorporating additional data, such as receptor status, is worthwhile to develop and update the chance algorithms. Partly because of its relatively recent introduction, data on HER2 remains relatively scarce. We have therefore interrogated our data on *BRCA1/2* primary mutation screens in women with invasive breast cancer and our *BRCA1* and *BRCA2* family register service databases to address the question of breast pathology in primary screens and of the proportion of *BRCA1* and *BRCA2* breast cancers that are triple negative or HER2 amplified [14]. We also subdivided HER2 amplified cancers by ER expression.

## Methods

Women with breast cancer have been tested for *BRCA1/2* mutations in Manchester since 1996. Data on women with breast cancer who had undergone *BRCA1/2* testing were obtained from those undergoing primary full screens for *BRCA1/2* mutations with sequencing of all exons and Multiple Ligation dependent Probe Amplification (MLPA) to assess for whole or multiple exon deletions or duplications as previously described [10]. Only women with confirmed pathogenic mutations were included as *BRCA1* or *BRCA2* positive. Whilst risk thresholds for testing were quite stringent with a 20% threshold for testing until 2013, the majority of women with at least a 10% probability for a *BRCA1/2* mutation and several at less than this had access to testing through research protocols.

All primary breast cancers occurring after 1990 (including contralateral) when hormone receptor testing started to be carried out routinely were included. HER2 testing did not become routine in the UK until around 2005. Data were collected prospectively on all women with breast cancer tested for *BRCA1/2* from 2005 and retrospectively for cases tested before that date. The study represents a semi-prospective consecutive series of women with breast cancer where HER2 status was assessable.

### Confirmation of HER2 positivity

*HER2* status was assessed from local pathology reports and defined as positive if (1)– scored 3+ by immunohistochemistry or (2) amplified by Fluorescent (or other) In Situ Hybridisation (ISH) with a HER2:CEP17 ratio of >2.0 or HER2 amplicon >6 if no CEP17 probe was employed. All negative, unconfirmed and borderline cases were excluded including those where tumours scored IHC 2+ from the pathology report and no supplementary FISH report was available.

### Confirmation of ER negativity

Breast cancers were classified as ER-ve based on pathology reports from clinical records and cancer registry data. ER was assessed as either a percentage staining (0–100%) or as a quickscore (QS) (0–8). A tumor was considered ER positive with a QS of 6–8 and or staining of >10%. ER negativity was confirmed if QS was <4 and or ER was <10%. Tumors with intermediate scores were excluded.

### Confirmation of triple negativity

From 2005, PR was also routinely tested and classification was identical to that of ER. PR positivity was not recorded on the mutation database but if ER and HER2 were negative and PR had a QS of <4 and or percentage was <10%, tumors were considered triple negative for the database.

Manchester scores were derived by summating scores for *BRCA1* and *BRCA2* for each breast, ovarian or prostate cancer primary in the same lineage. An adjustment of −4 points was made for a HER2+ breast cancer and +4 for a grade 3 triple negative breast cancer as previously described [10]. Two sided Chi-square tests with Fisher’s exact correction were used to compare proportions.

Ethics approval for the study was through the North Manchester Research (08/H1006/77) and University of Manchester ethics committees (08229).

## Results

There were 1134 women with breast cancer with verified HER2 and ER status who had undergone full mutation screening of *BRCA1* and *BRCA2* (Table 1). Included were 619 of the total of 803 (77%) samples that had BRCA tested in Manchester in the last 4 years (since 01/11/2011). Twenty-three women with breast cancer were excluded from the main analysis as their tumours were ER negative and PR positive, resulting in 1111 eligible women with ER/PR negative tumour with a known HER2 status. The majority (n = 101) of the 184 women in the most recent era (2010–2015) without HER2 status were diagnosed with breast cancer before 2005, 78 (9.8%) had no available pathology report and the remaining five (0.6%) were HER2 2+ without available FISH testing from clinical notes. Of the 1111 women, 161 (14.5%) had *HER2* amplified breast cancer. 114/161 (71%) were ER positive. Only 9/161 (5.6%) of BRCA tests in HER2+ women identified a pathogenic mutation. Five of 114 (4.4%) of ER+ HER2+ cancers had mutations (4 *BRCA2*) and 4/45 (9%) of ER− HER2+ (3 *BRCA1*). In contrast, of 425 screens in women with triple negative breast cancer, 151 (35.5%) resulted in positive BRCA tests with 117 (27.5%) having *BRCA1* mutations and 34 (8.0%) *BRCA2* mutations. Even with a combined pathology adjusted Manchester score [10] of 20 or higher indicating at least a 20% likelihood of a *BRCA1/2* mutation only 5/33 (15%) women with HER2+ breast cancer had a mutation identified. However, this was 4/10 (40%) of ER− HER+ cancers and only 1/23 (4.4%; 95% CI 0–12.7%) of ER+ HER2− cancers (*p* = 0.02). It was not really possible to assess the 10% threshold with a Manchester score of 15–19 as only seven breast cancers that were ER− HER2+ were tested one of which (14%) had a *BRCA2* mutation. None of 22 sporadic HER2+ breast cancers had a *BRCA1/2* mutation, but 2/13 (15%) diagnosed <35 years had a *TP53* mutation. In contrast 120/215 (55.8%) of those with triple negative breast cancer and a Manchester score above 20 had a mutation identified, rising to 83% in women with a Manchester score of 30 or higher.

**Table 1:** HER2 and ER status in primary BRCA screens

	*BRCA1*	*BRCA2*	negative	Total	Proportion with *BRCA* mutations (%)	95% Confidence intervals (%)
HER2+ MS < 14	0	1	81	82	1.2	0.0–3.6
HER2+ MS 14–20	1	2	43	46	6.5	0.0–13.7
ER+ HER2+ 14–20	1	1	37	39	5.1	0.0–12.1
HER2+ MS 20+	3	2	28	33	15	2.9–27.4
ER+ HER2+ MS 20+	0	1	22	23	4.4	0–12.7
Total HER2+	4	5	152	161	5.6	2.0–9.2
ER− HER2− <50 years	103	25	213	341	37.5	32.4–42.7
ER− HER2− 50+	14	9	61	84	27.3	17.8–36.9
ER− HER2− sporadic < 50 years	6	2	86	94	8.5	2.9–14.2
ER− HER2− MS < 14	6	0	79	85	7.0	1.6–12.5
ER− HER2− MS 14–20	15	10	100	125	20.0	13.0–27.0
ER− HER2− MS 20+	96	24	95	215	55.8	49.2–62.4
ER− HER2− MS 30+	51	8	12	71	83.1	74.4–91.8
Total ER− HER2−^a^	117	34	274	425	35.5	30.9–40.0

^a^This excludes 23 women with ER− PR+ HER2− breast cancers. None had *BRCA1/2* mutations

*MS* manchester score

Interestingly, the 10% threshold was not met with triple negative breast cancers with a Manchester score of <14 (6/85 = 7.0%), nor with sporadic triple negative cases of breast cancer aged <50 years at diagnosis (8/94 = 8.5%).

## Discussion

This present study has demonstrated a very low frequency of detection of *BRCA1* and *BRCA2* mutation carriers amongst primary screens of women with HER2 amplified breast cancers, particularly those with ER+ ve tumours. Overall, these low rates do not appear to be fully accounted for in the pathology adjusted Manchester scoring system with the 10% risk combined threshold not being met in women with scores of 14–19 and the 20% threshold not being met in women with Manchester scores of 20 or higher (5/33–15%), excepting those that were ER-ve. In practice, this suggests that women with ER+ HER2 positive breast cancers should be advised that they are unlikely to harbour a *BRCA1/2* mutation unless there are other very suggestive features in their own personal or family histories (other more typical breast cancer or ovarian cancer). Additionally, women who are offered testing whose mother or sister had a HER2 positive breast cancer will get little reassurance regarding breast cancer risk from a negative test unless there is also a strong additional family history suggestive of *BRCA1/2*. This is because these women will still have an increased risk of breast cancer, due to the likely presence of other breast cancer genes within their families.

Whilst the low level of detection of *BRCA1* and *BRCA2* mutations amongst individuals with HER2+ breast cancers is clearly important in assessing carrier likelihood, the presence of a triple negative breast cancer clearly increases the likelihood of identifying a *BRCA1/2* mutation. It has been suggested that all women with triple negative breast cancer aged <50 years should be tested for *BRCA1/2* mutations based on the overall detection rate being above 10% [15, 16]. However, this does not take into account the fact that the 10% threshold is not clearly reached in women with an isolated breast cancer and no family history [15], In a large study of 1824 cases [15], only 18/209 (8.6%) women with sporadic triple negative breast cancer diagnosed aged 40–49 years had an identifiable mutation in *BRCA1/2.* This is supported by a detection rate of only 8/94 (8.5%) in sporadic triple negative cases <50 years in the present study although the 95% confidence intervals do overlap with 10–8.5% (95% CI 2.9–14.2%). Nonetheless, sporadic cases under 40 years do meet the 10% threshold [17] with 23/149 (18%) of 35–39 year old sporadic cases having a mutation and 23% (18/91) of those <35 years of age at diagnosis. Clearly 8.6% is still close to the 10% threshold (the upper 95% CI is 12.4%) and many centres may consider it simpler just to test all cases <50 years. Additionally, testing women with few unaffected female relatives and in particular, adopted women seems appropriate as the 10% threshold may be reached in these groups.

At the other end of the spectrum, unaffected women whose mother or sister with high grade triple negative breast cancer is unavailable for testing and whose family history is strongly predictive of a *BRCA1/2* mutation, (such as a BOADICEA likelihood in the relative of >80% or a pathology adjusted Manchester score of 30 or higher) should be reassured by a negative test. The majority of their inherited risk would be due to a discoverable *BRCA1/2* mutation and the negative test will greatly reduce their risk of breast and ovarian cancer, due to the high sensitivity of current BRCA testing [18].

**Fig. 1:**
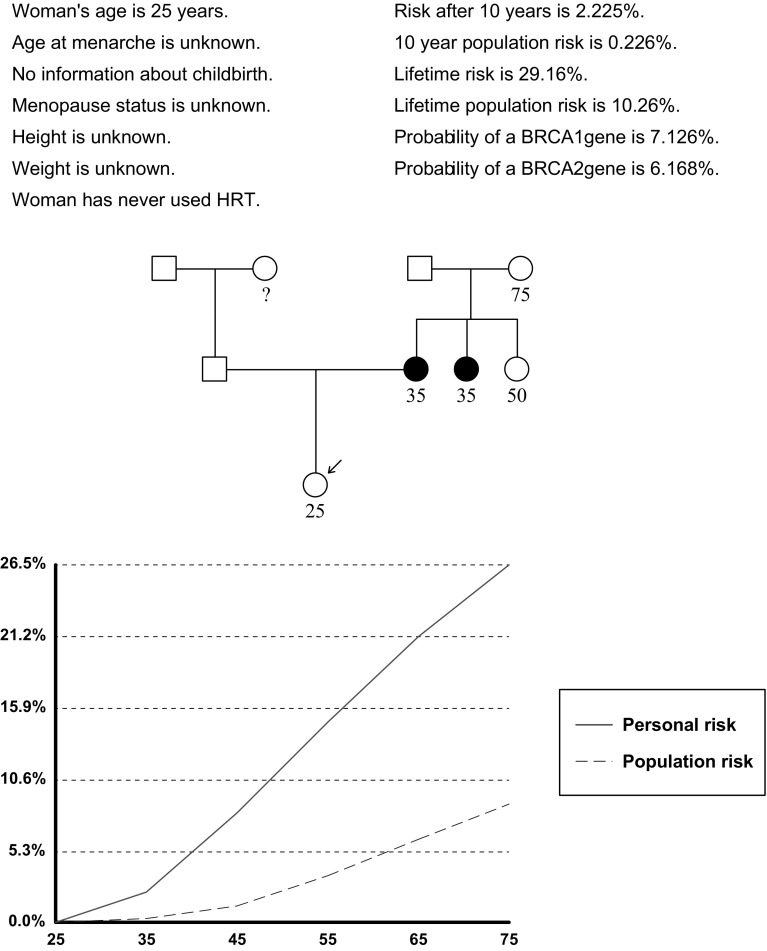
Tyrer-Cuzick risk readout of unadjusted breast cancer risk estimation of case example

An example pedigree (Fig. 1) is given to show the effects of using or not using HER2 status in assessing breast cancer risk for a 25-year old unaffected woman, with an affected mother and maternal aunt with breast cancers at age 35 years. Using the Manchester score [10], a grade 3 triple negative breast cancer would add 4 points to the Manchester score of 16 to reach 20 points. A HER2+ breast cancer in the mother would reduce the score to 12 points. An unadjusted Manchester score would be equivalent to a 10% probability of *BRCA1/2* in the mother. This would rise to 20% with Triple negative and reduce to 5% if mother had a HER2+ tumour. The likelihoods are halved in the proband. The attributable risks of breast cancer using 80% penetrance would be only 2% if mother was HER2+ rising to 8% if mother was triple negative (Table 2). Readouts for Tyrer-Cuzick (changes to BRCA1/2 probability inferred) and BOADICEA [12] and BRCAPRO [5] (both with inbuilt pathology adjustments) are shown in Table 2. Apart from with Tyrer-Cuzick, which does not have an inbuilt adjustment for pathology, the reduction in breast cancer risk is only about 2% for testing negative in the proband when the mother was HER2+. This changes to a 5.7–10.5% reduction if the mother had triple negative breast cancer. With BOADICEA the *BRCA1/2* probability in the proband falls from 6.5 to 3.6% with HER2+ breast cancer in mother and rises to 12.3% with triple negative. A negative *BRCA1/2* mutation test only drops absolute breast cancer risk by 1.7% if mother was Her2+ but by nearly 6% when triple negative. These may underestimate the reductions due to the default BRCA sensitivities being only 70 and 80% for *BRCA1/2* respectively, which is below the sensitivity of at least 84% (Table 1) for triple negatives with Manchester scores above 30 in this report and our previous identification of *BRCA1/2* mutations in 81/94 (86%) of breast/ovarian families with Manchester scores of 40+ [19]. Whilst the downward adjustment for ER− HER2+ breast cancers in BOADICEA appears appropriate, the programme currently does not adjust for HER2+ breast cancers when they are also ER+. The downward adjustment for an ER+ HER2+ breast cancer is only from 6.5 to 4.7% whereas the current report only identified mutations in 4.5% of ER+ HER2+ breast cancers when the average detection rate in ER+ HER2− breast cancer was 14.8% (Table 1). The current data would therefore suggest that further downward adjustment for HER2 amplification is still necessary for ER+ tumours with BOADICEA. Using BRCAPRO (from Cagene v.6) there is a greater adjustment with a 10% likelihood in the proband for BRCA1/2 dropping to 3.4% with HER2+ in mother and rising to 22.5% with triple negative. However, the breast cancer risk readouts for BRCAPRO only include familial risk from *BRCA1/2* and therefore the reassurance of reducing risks to population levels after negative *BRCA1/2* testing is inappropriate, [20] as is demonstrated by the far lower breast cancer risk predictions with BRCAPRO compared to Tyrer-Cuzick and BOADICEA. BRCAPRO significantly underestimates breast cancer risk in the familial breast cancer risk setting [20]. Nonetheless BRCAPRO does have a specific readout for ER+ HER2+ breast cancer that is different to ER− HER2+ of 4.8%. Overall an approximate halving of BRCA probability with a HER2+ breast cancer and doubling with a triple negative breast cancer appears to fit the current data.

**Table 2:** Change in breast cancer risks given to a woman with mother and maternal aunt with breast cancer in their thirties when mother had an ER− Her2+ or grade 3 triple negative (G3TN) breast cancer based on *BRCA1/2* testing of the unaffected proband

	Risk to 80 years of age prior to *BRCA1/2* testing Not taking into account histology	Adjusted risk taking into account change in *BRCA1/2* probability from histology prior to testing	Final risk based on negative BRCA1/2 testing	Reduction in breast cancer risk based on negative testing (%)
Tyrer-Cuzick v.6 Her2+	*BRCA1/2* 13% 29% BC risk	*BRCA1/2* 6.5%^b^ 24% BC risk	*BRCA1/2* 0.5%^a^ 19% BC risk	5
Tyrer-Cuzick v.6 G3TN	*BRCA1/2* 13% 29% BC risk	*BRCA1/2* 26%^b^ 39% BC risk	*BRCA1/2* 2%^a^ 19% BC risk	20
BOADICEA HER2+^c^	*BRCA1/2* 6.5% 26% BC risk	*BRCA1/2* 3.6% 24% BC risk	*BRCA1/2* 1%^c^ 22.3% BC risk	1.7
BOADICEA G3TN	*BRCA1/2* 6.5% 26% BC risk	*BRCA1/2* 12.3% 29.5% BC risk	*BRCA1/2* 3.83% 23.7% BC risk^c^	5.8
BRCAPRO Her2+	*BRCA1/2* 10% 17% BC risk	*BRCA1/2* 3.4% 13.7% BC risk	*BRCA1/2* 0.2% 12.2% BC risk	1.5
BRCAPRO G3TN	*BRCA1/2* 10% 17% BC risk	*BRCA1/2* 22.5% 23.3% BC risk	*BRCA1/2* 1.2% 12.8% BC risk	10.5
Breast cancer risk attributable from *BRCA1/2* alone using Manchester score Her2+	10% likelihood in mother with MS 16 4% BC risk	5% likelihood in mother with MS 12 2% BC risk		2
Breast cancer risk attributable from *BRCA1/2* alone using Manchester score G3TN	10% likelihood in mother with MS 16 4% BC risk	20% likelihood in mother with MS 20 8% BC risk		8

BRCAPRO readout from Cagene v.6 https://www4.utsouthwestern.edu/breasthealth/cagene/

*G3TN* grade 3 triple negative

^a^This assumes >90% sensitivity of *BRCA1/2* testing

^b^This assumes a halving of *BRCA1/2* probability with Her2+ and doubling with G3TN and no change of BRCAX status

^c^Although the current online BOADICEA (https://pluto.srl.cam.ac.uk/) does include HER” and ER status HER” is only taken into account when ER is negative. BRCA1/2 testing sensitivities are set at 70 and 80% for BRCA1/2

The present study does have some limitations. The numbers are relatively small compared to large consortia [14], but this allows consistency of the approach to classifying HER2 status. In recent times testing of HER2+ samples will have been relatively reduced because of implementing a pathology adjusted Manchester score [10]. This will have boosted numbers with triple negative cancers at the expense of HER2+ cancers thus reducing the overall rate with HER2+ cancers to 13.5% which is below the overall rate in all breast cancers. It is not clear whether HER2+ breast cancers are more or less likely to have a familial component outside of *BRCA1* and *BRCA2,* although *TP53* related breast cancers, which make up a very small proportion of familial breast cancer, are usually HER2+ [21, 22]. Indeed one aspect of the present study is that sporadic HER2+ breast cancer is extremely unlikely to have a *BRCA1/2* mutation but <35 years may well have a *TP53* mutation. With more women with breast cancers at early ages undergoing mutation testing to determine treatment even without a family history, extra weight should be given to discussing *TP53* in very young sporadic HER2+ breast cancers than the very small possibility of *BRCA1/2*. Although PR was not collected systematically it was when both ER and HER2 were negative. In a large study of 631 breast cancers that were HER2− and ER− only 43 (6.8%) were PR positive [23] similar to the 23/438 (5.2%) in the current study.

In conclusion the present study demonstrates the great importance of properly assessing breast cancer HER2 status when determining the likelihood of a *BRCA1/2* mutation. Where possible this information should be sought, especially when testing unaffected women whose affected relative with breast cancer is unavailable for genetic testing. Use of well validated programmes that take into account the possibility of familial risk other than *BRCA1/2* should be used although further adjustments may need to be made before these models fully account for the effects of HER2+ status.
